# PD-1/PD-L1 and coronary heart disease: a mendelian randomization study

**DOI:** 10.3389/fcvm.2024.1424770

**Published:** 2024-10-18

**Authors:** Liangjia Zeng, Yinglan Liang, Ruoyun Zhou, Wenting Yang, Kexin Chen, Baixin He, Yuqing Qiu, Linglong Liu, Deyang Zhou, Zhaolin Xiao, Haowen Liang, Binghua Zhang, Renyu Li, Lihong Yu, Min Yi, Xiaozhen Lin

**Affiliations:** ^1^ The Second Affiliated Hospital, Guangzhou Medical University, Guangzhou, China; ^2^ Department of Clinical Medicine, Clinical Medical School, Guangzhou Medical University, Guangzhou, China; ^3^ Medical Exploration and Translation Team, Cardiovascular Medicine and Cardio-Oncology Group, Guangzhou, China; ^4^ Department of Anesthesiology, Clinical Medical School, Guangzhou Medical University, Guangzhou, China; ^5^ Department of Medical Imageology, Clinical Medical School, Guangzhou Medical University, Guangzhou, China; ^6^ Department of Psychiatry, Clinical Medical School, Guangzhou Medical University, Guangzhou, China; ^7^ School of Pharmaceutical Sciences, Guangzhou Medical University, Guangzhou, China; ^8^ Department of Endocrinology, The Second Affiliated Hospital of Guangzhou Medical University, Guangzhou, China; ^9^ Department of Cardiology, Guangzhou Institute of Cardiovascular Disease, Guangdong Key Laboratory of Vascular Diseases, The Second Affiliated Hospital, Guangzhou Medical University, Guangzhou, China

**Keywords:** Mendelian randomization (MR), coronary heart disease (CHD), PD-1, PD-L1, GSEA

## Abstract

**Introduction:**

It has been found that programmed cell death protein-1 (PD-1) or its ligand PD-L1 may play an important role in the onset and progression of coronary heart disease (CHD). Thus, we conducted this mendelian randomization analysis (MR) to estimate the causal relationship between PD-1/PD-L1 and 5 specific CHDs (chronic ischemic heart disease, acute myocardial infarction, angina pectoris, coronary atherosclerosis, and unstable angina pectoris), complemented by gene set enrichment analysis (GSEA) for further validation.

**Methods:**

Publicly available summary-level data were attained from the UK Biobank with genetic instruments obtained from the largest available, nonoverlapping genome-wide association studies (GWAS). Our analysis involved various approaches including inverse variance-weighted meta-analysis, alternative techniques like weighted median, MR-Egger, MR-multipotency residuals and outliers detection (PRESSO), along with multiple sensitivity assessments such as MR-Egger intercept test, Cochran's Q test, and leave-one-out sensitivity analysis to evaluate and exclude any anomalies.

**Results:**

Gene expression profile (GSE71226) was obtained from Gene Expression Omnibus (GEO) database for GSEA. IVW analysis showed a causal association between PD-1 and chronic ischemic heart disease (OR, 0.997; 95%CI, 0.995-0.999; *P*, 0.009), chronic ischemic heart disease and PD-1 (beta, −3.1; 95%CI, −6.017 to −0.183; *P*, 0.037), chronic ischemic heart disease and PD-L1 (beta, −3.269; 95%CI, −6.197 to −0.341; *P*, 0.029). No significant causal relationship was found between PD-1/PD-L1 and other 4 CHDs. The accuracy and robustness of these findings were confirmed by sensitivity tests. GSEA found that the KEGG pathway and related core genes of “PD-L1 expression and PD-1 checkpoint pathway in cancer” pathway were downregulated in CHD.

**Discussion:**

This study provided evidence of a bidirectional causal relationship between PD-1 and chronic ischemic heart disease and a protective association between chronic ischemic heart disease and PD-L1.

## 1 Introduction

Coronary heart disease (CHD) remains the leading cause of death worldwide (1). According to statistics released by the World Health Organization (WHO), CHD accounts for 16% of total deaths in the world, ranking as the foremost cause of death of the top 10 most deadly diseases globally. Since 2000, the number of deaths CHD deaths has increased significantly, with 2 million more deaths in 2019, totaling 8.9 million (2).

The basic pathological basis for the development of CHD is coronary atherosclerosis formation and plaque deposition, where inflammation and immune response have been shown to play an important role (3, 4). Previous studies have focused on cancer patients, claiming that PD-1/PD-L1 inhibitors accelerate atherosclerosis in cancer patients (5, 6). The role of PD-1/PD-L1 in the development of CHD in the general population needs to be further investigated. Some studies in mice have shown that low expression of PD-1/PD-L1 or anti-PD-1/PD-L1 treatment exacerbated atherosclerotic plaque formation and accelerated the immune process of CHD (7, 8). Clinical studies in patients with atherosclerosis have shown that abnormal PD-1/PD-L1 expression is associated with CHD (9, 10). Nevertheless, no trustworthy evidence exists to support the therapeutic effect of either upregulating or inhibiting the PD-1/PD-L1 pathway in CHD patients (11). Considering the limitations of different immune backgrounds, small sample sizes, single centers, and other confounding factors, previous studies can only suggest an association of PD-1/PD-L1 deficiency or low expression with CHD, rather than establishing a definitive causal relationship between PD-1/PD-L1 and CHD.

Mendelian randomization (MR) is an important method widely used in epidemiology for the assessment of potential causal relationships between exposure factors and clinical disease. The independent segregation of alleles at conception means that they are not affected by potential confounders, forming a natural experiment similar to a randomized trial, and thus Mendelian randomization analyses may provide more reliable insights into the potential causal relationship between PD-1/PD-L1 and CHD than traditional observational analyses (12–14). Gene set enrichment analysis (GSEA) is a powerful tool to associate a disease phenotype to a group of genes/proteins. Therefore, we conducted a mendelian randomized study to explore the causal relationship between PD-1/PD-L1 and five specific types of CHD (chronic ischemic heart disease, acute myocardial infarction, angina pectoris, coronary atherosclerosis, and unstable angina pectoris), complemented by GSEA for further validation.

## 2 Materials and methods

### 2.1 Study design

MR uses genetic instrumental variables (IVs) to evaluate the causal relationship between exposure and outcome. The basic principle of MR design is that genetic variations are fixed at conception and randomly assigned to individuals. Thus, it can overcome problems of unmeasured confounding and reverse causation typical of conventional observational epidemiology. In two sample MR, it combines data from multiple sources and uses two different research samples to estimate instrumental risk factors and instrumental outcome associations.

MR should be performed under three basic assumptions: (1) genetic variation is strongly associated with exposure; (2) genetic variation is independent of any potential confounding factors; and (3) genetic variation is independent of outcome except by means of exposure.

The design of our study had three key components: (1) the identification of genetic variants to serve as IVs; (2) the estimation of overall causal effect utilizing two sample, multivariable and bidirectional MR strategy; (3) the assessment of horizontal pleiotropy and validation of results conducting sensitivity analysis.

We further performed Gene Set Enrichment Analysis (GSEA) to further consolidate the causal relationship detected, checking whether CHD is associated with genes related to PD-1/PD-L1 pathway.

All relevant data we used are publicly downloadable on the website. All of these data are anonymous, freely downloadable, and can be used without restriction.

### 2.2 Data source

#### 2.2.1 Data for exposure

The exposure of this study is defined as the plasma level of PD-1 and PD-L1. We used the summary-level data source from a genome-wide test of 10.6 million imputed autosomal variants against levels of 2,994 plasma proteins in 3,301 individuals of European descent, whose number of SNP was 10,534,735 (GWAS ID: prot-a-2214 and prot-a-431). This GWAS employed a complex method using special aptamers to measure the levels of 3,622 plasma proteins or protein complexes with 4,034 modified aptamers. The assay enhances the detection threshold for protein abundance beyond what traditional methods, such as immunoassays, typically allow. It is capable of measuring both extracellular and intracellular proteins, including the soluble domains of proteins associated with membranes (15). Detailed information is shown in Supplementary Table S1.

#### 2.2.2 Data for outcome (Supplementary Table S1)

The outcome is defined as 5 self-reported doctor-diagnosed CHD: chronic ischemic heart disease (ICD10: I25), acute myocardial infarction (ICD10: I21), angina pectoris (ICD10: I20), coronary atherosclerosis, unstable angina pectoris. The GWAS summary statistics of chronic ischemic heart disease, acute myocardial infarction and angina pectoris were extracted from Neale lab analysis of UK Biobank phenotypes, round 1 (http://www.nealelab.is/blog/2017/7/19/rapid-gwas-of-thousands-of-phenotypes-for-337000-samples-in-the-uk-biobank). In particular, The GWAS summary statistics of chronic ischemic heart disease (GWAS ID: ukb-a-534) contained 8,755 cases and 328,444 controls; The GWAS summary statistics of acute myocardial infarction (GWAS ID: ukb-a-533) included 3,927 cases and 333,272 controls; The GWAS summary statistics of angina pectoris (GWAS ID: ukb-a-532) included 4,837 cases and 332,362 controls. Meanwhile, we extracted data on associations of relevant SNPs with coronary atherosclerosis and unstable angina pectoris from the Neale lab analysis of UK Biobank phenotypes, round 2 (http://www.nealelab.is/uk-biobank/). The GWAS summary statistics of coronary atherosclerosis (GWAS ID: ukb-d-I9_CORATHER) was based on 361,194 samples with 13,586,589 SNPs, including 14,334 cases and 346,860 controls. The GWAS summary statistics of unstable angina pectoris (GWAS ID: ukb-d-I9_UAP) were derived from 361,194 samples with 11,385,655 SNPs, including 3,439 cases and 357,755 controls.

#### 2.2.3 Data for GSEA

Expression data set (GSE71226) was obtained from Gene Expression Omnibus (GEO) database, including 3 patients with coronary heart disease and 3 healthy people. Total RNA of each samples were extracted from peripheral blood to hybridize with Affymetrix microarrays (https://www.ncbi.nlm.nih.gov/geo/).

### 2.3 Selection of instrumental variables (IVs)

The effect allele for each SNP was defined as the allele associated with increased level of the relevant exposure. The genetic IVs were obtained based on the following three criteria: (1)For standard Mendelian randomization, single-nucleotide polymorphisms (SNPs) associated with the exposure, as well as the reverse study, were selected at the genome-wide significance level (*p* ≤ 5 × 10–6); (2)SNPs were clumped based on linkage disequilibrium (r2 < 0.001 and kB = 10,000); (3)Duplicate SNPs and palindromic SNPs were removed.

We also calculated the conditional F statistic to characterize instrument strengths. For a single variant, the F statistic is equal to the square of the genetic association with the exposure divided by the square of its standard deviation, calculated by the formula F = *β*i2/se(*β*i)2 and a value ≥ 10 was considered sufficient (16). All selected SNP were with F statistic ≥10.

### 2.4 Statistical analysis

#### 2.4.1 Univariable MR

Inverse-variance weight (IVW) were the principal analyses for our study as it is the most efficient analysis method with valid instrumental variables, accounting for heterogeneity in the variant-specific casual estimates. This method provided a high-powered estimate and relied on the assumption that all SNPs were valid genetic instruments (17).

For sensitivity analysis, we performed multiple methods including Mendelian randomization-Egger (MR-Egger), Robust adjusted profile score (RAPS), the weighted median approach, the Mendelian Randomization Pleiotropy RESidual Sum and Outlier (MR-PRESSO), Cochran's Q statistic, leave-one-SNP-out analysis, and horizontal pleiotropy analysis.

The MR-Egger method offers a relatively stable estimate, independent of the instrumental variables’ validity, and adjusts for potential horizontal pleiotropy through regression slope and intercept adjustments (17). We calculated the intercept of MR-Egger to provide a measurement of horizontal pleiotropy, confirming if the variant has a direct effect on the target outcome (18).

Robust adjusted profile score (RAPS) is a recently recommended method which is robust to both systematic and idiosyncratic pleiotropy and can give a robust inference for MR analysis with many weak instruments (19).

The weighted median approach gives consistent estimates of the causal effect under the assumption that genetic variants representing over 50% of the weight in the analysis are valid instruments. The method is provided to ensure the median of all the instrumental variable estimates based on the individual genetic variants will be a consistent estimate (20).

MR-PRESSO method was used to test, and correct, if needed, for possible horizontal pleiotropic outliers in the analysis by removing SNPs that contribute to the heterogeneity disproportionately more than expected (18).

Cochran's Q statistic is a statistical test for heterogeneity which is derived from the IVW estimate, which follows ×2 distribution with degrees of freedom equal to the number of SNPs minus 1.

Leave-one-SNP-out analysis may provide us a dominant estimate of the casual effect when there is one genetic variant that is particularly strongly associated with the exposure.

#### 2.4.2 Multivariable MR

The Multivariable Mendelian Randomization technique is suitable for employing numerous genetic instruments irrespective of their linkage to the exposure (21). While the instrumental variables might be linked to multiple risk factors, they must meet the equivalent instrumental-variable assumptions. Consequently, we utilized this approach, incorporating all instrumental variables pertaining to PD-1 and PD-L1, to ascertain their distinct impacts on CHD, employing IVW as our primary analytical method and MR-Egger as a sensitivity analysis.

#### 2.4.3 Bidirectional MR

We additionally performed Bidirectional Mendelian randomization to assesses the effect of the outcome on the exposure (22), aiming to clarify whether the presence of CHD has a negative effect on PD-1/PD-L1, or whether the correlation between the two is due to latent confounding. The primary analyses for our bidirectional MR were IVW with multiple methods (MR-Egger, RAPS, the weighted median approach, MR-PRESSO, Cochran's Q statistic, leave-one-SNP-out analysis, horizontal pleiotropy analysis) performed as sensitivity analysis.

#### 2.4.4 GSEA analysis

Initially, the Limma R package was employed to determine the log2(fold change) and *P* values for the expression data set. Subsequently, using clusterProfiler and org.Hs.eg.db R package, Gene Set Enrichment Analysis (GSEA) was performed to identify the core differentially expressed genes (DEGs), utilizing the KEGG gene set as the predefined set for enrichment analysis. Criteria for selecting significant enrichment pathways associated with coronary disease traits were established at a *P*-value of less than 0.05 and a false discovery rate (FDR) q value of less than 0.25.

## 3 Results

### 3.1 Univariable MR

**Figure 1:**
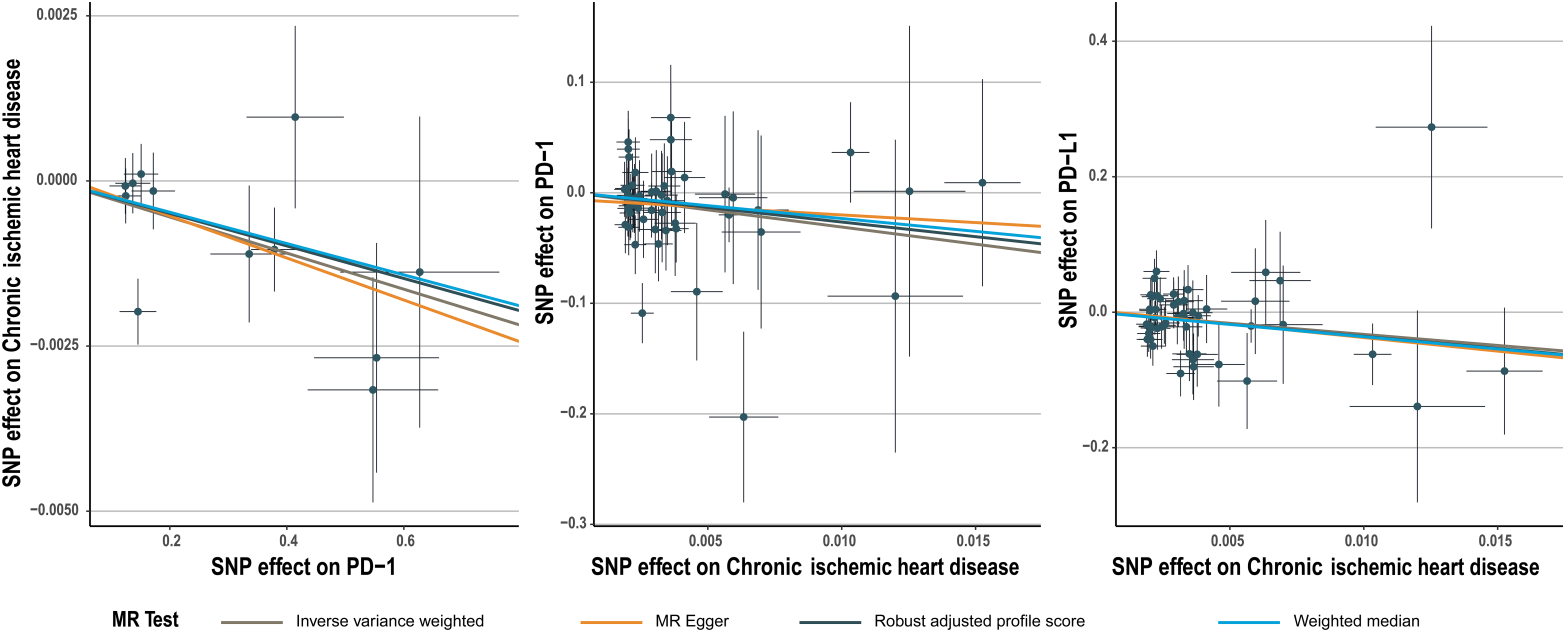
Scatter plot for PD-1 effect on chronic ischemic heart disease and chronic ischemic heart disease effect on PD-1/PD-L1.

The number of SNPs selected for PD-1 or PD-L1 ranged from 6 to 13. All SNPs selected for inclusion in univariable MR analysis are presented in Supplementary Table S2–S11. The IVW analysis found clear evidence of a protective causal effect of PD-1 on chronic ischemic heart disease (OR, 0.997; 95%CI, 0.995–0.999; *P*, 0.009), which was replicated by RAPS and WM (Table 1). MR-Egger analysis also showed a protective but not significant effect of PD-L1 on the risk of myocarditis (Table 1). In addition, there was no evidence in favour of an association between PD-1 and other four types of CHD, including acute myocardial infarction, angina pectoris, unstable angina pectoris, and coronary atherosclerosis (Table 1). We found no statistically significant effect of PD-L1 on the 5 types of CHD mentioned above (Table 2). No outlier between PD-1/PD-L1 and the risk of myocarditis was identified by the MR-PRESSO test (Tables 1 and 2). No directional pleiotropy was found in the MR-Egger regression (Tables 1 and 2). No heterogeneity, except for estimates of effect of PD-1 and PD-L1 on coronary atherosclerosis, was found by Cochran's Q statistic (Tables 1 and 2). The scatter plot and leave-one-out plot for PD-1/PD-L1's effects on the 5 CHD mentioned above are available in Figure 1, Supplementary Figures S1, S2, S7, S8.

**Table 1:** Single-variable MR results of PD-1 on risk of chronic ischemic heart disease, acute myocardial infarction, angina pectoris, unstable angina pectoris and coronary atherosclerosis.

Outcome	N SNP	Methods	OR (95% CI)	*P*-value	Q-statistics	*P*h	*P*p
Chronic ischemic heart disease	12	IVW	0.997 (0.995–0.999)	0.009*	16.414	0.13	
		MR Egger	0.997 (0.992–1.001)	0.18	16.326	0.09	0.82
		RAPS	0.998 (0.996–0.999)	0.009*			
		Weighted median	0.998 (0.995–0.999)	0.043*			
		MR Presso	NA	NA			
Acute myocardial infarction	12	IVW	1.000 (0.998–1.001)	0.83	19.007	0.06	
		MR Egger	1.001 (0.998–1.004)	0.49	17.428	0.07	0.36
		RAPS	1.000 (0.999–1.002)	0.82			
		Weighted median	1.001 (0.999–1.002)	0.49			
		MR Presso	NA	NA			
Angina pectoris	12	IVW	1.000 (0.999–1.001)	0.73	6.504	0.84	
		MR Egger	0.999 (0.997–1.002)	0.67	6.024	0.81	0.50
		RAPS	1.000 (0.999–1.002)	0.74			
		Weighted median	1.000 (0.998–1.002)	0.94			
		MR Presso	NA	NA			
Unstable angina pectoris	13	IVW	1.000 (0.999–1.001)	0.52	10.854	0.54	
		MR Egger	1.000 (0.998–1.002)	0.87	10.574	0.48	0.61
		RAPS	1.000 (0.999–1.001)	0.46			
		Weighted median	1.000 (0.999–1.002)	0.69			
		MR Presso	NA	NA			
Coronary atherosclerosis	13	IVW	0.998 (0.995–1.001)	0.19	29.53	0.003	
		MR Egger	0.999 (0.993–1.006)	0.82	29.103	0.002	0.70
		RAPS	0.998 (0.996–1.001)	0.20			
		Weighted median	0.999 (0.996–1.001)	0.29			
		MR Presso	0.999 (0.997–1.001)	0.35			

IVW, inverse variance weighted; RAPS, robust adjusted profile score; MR Presso, Mendelian randomization pleiotropy RESidual sum and outlier; N SNP, number of genetic instruments; OR, odds ratio; CI, confidence interval; *P*h, *P*-value for heterogeneity; *P*p, *P*-value for Pleiotropy; NA, not applicable.

*P < 0.05.

**Table 2:** Single-Variable MR results of PD-L1 on risk of chronic ischemic heart disease, acute myocardial infarction, angina pectoris, unstable angina pectoris and coronary atherosclerosis.

Outcome	SNPS	Methods	OR (95% CI)	*P*-value	Q-statistics	*P*h	*P*p
Chronic ischemic heart disease	6	IVW	1.000 (0.997–1.002)	0.70	4.961	0.42	
		MR Egger	1.003 (0.992–1.015)	0.61	4.494	0.34	0.55
		RAPS	1.000 (0.997–1.002)	0.71			
		Weighted median	1.001 (0.998–1.004)	0.57			
		MR Presso	NA	NA			
Acute myocardial infarction	6	IVW	0.999 (0.997–1.000)	0.16	3.989	0.55	
		MR Egger	1.001 (0.993–1.008)	0.88	3.757	0.44	0.66
		RAPS	0.999 (0.997–1.001)	0.18			
		Weighted median	0.998 (0.996–1.000)	0.12			
		MR Presso	NA	NA			
Angina pectoris	6	IVW	1.000 (0.998–1.002)	0.87	2.925	0.71	
		MR Egger	0.999 (0.991–1.008)	0.88	2.909	0.57	0.90
		RAPS	1.000 (0.998–1.002)	0.87			
		Weighted median	1.000 (0.998–1.002)	0.84			
		MR Presso	NA	NA			
Unstable angina pectoris	8	IVW	1.000 (0.999–1.001)	0.77	4.535	0.72	
		MR Egger	0.999 (0.993–1.004)	0.64	4.196	0.65	0.58
		RAPS	1.000 (0.999–1.001)	0.82			
		Weighted median	1.000 (0.999–1.002)	0.70			
		MR Presso	NA	NA			
Coronary atherosclerosis	8	IVW	0.998 (0.995–1.002)	0.40	14.545	0.04	
		MR Egger	1.011 (0.998–1.024)	0.14	8.664	0.19	0.09
		RAPS	0.999 (0.995–1.003)	0.64			
		Weighted median	1.000 (0.997–1.004)	0.81			
		MR Presso	NA	NA			

IVW, inverse variance weighted; RAPS, robust adjusted profile score; MR Presso, Mendelian randomization pleiotropy RESidual sum and outlier; N SNP, number of genetic instruments; OR, odds ratio; CI, confidence interval; *P*h, *P*-value for heterogeneity; *P*p, *P*-value for Pleiotropy; NA, not applicable.

### 3.2 Multivariable MR

In the multivariable MR that adjusted for the effect of PD-1 and PD-L1, the strong negative association between PD-1 and chronic ischemic heart disease remained and the association with other 4 types of CHD was still not significant. The multivariable-adjusted OR was 0.998 (95CI%, 0.996–0.999; *P*, 0.015) for association between PD-1 and chronic ischemic heart disease (Table 3). The causal relationship between PD-L1 and the 5 CHD remained insignificant (Table 3). The selected SNPs were listed in Supplementary Table S12–S16.

**Table 3:** Multivariable MR results of PD-1/PD-L1 on risk of chronic ischemic heart disease, acute myocardial infarction, angina pectoris, unstable angina pectoris and coronary atherosclerosis.

Exposure	Outcome	SNPS	Methods	OR (95% CI)	*P*-value
PD-1	Chronic ischemic heart disease	18	IVW	0.998 (0.996–0.999)	0.02*
			MR Egger	0.997 (0.994–1.000)	0.06
PD-L1	Chronic ischemic heart disease	18	IVW	0.999 (0.996–1.002)	0.50
			MR Egger	0.999 (0.996–1.002)	0.61
PD-1	Acute myocardial infarction	18	IVW	1.000 (0.999–1.001)	0.94
			MR Egger	1.000 (0.998–1.002)	0.90
PD-L1	Acute myocardial infarction	18	IVW	0.998 (0.997–1.000)	0.09
			MR Egger	0.998 (0.997–1.000)	0.12
PD-1	Angina pectoris	18	IVW	1.000 (0.999–1.002)	0.68
			MR Egger	1.000 (0.998–1.002)	0.72
PD-L1	Angina pectoris	18	IVW	1.000 (0.998–1.002)	0.88
			MR Egger	1.000 (0.999–1.002)	0.72
PD-1	Unstable angina pectoris	21	IVW	1.000 (0.999–1.001)	0.47
			MR Egger	1.000 (0.999–1.002)	0.71
PD-L1	Unstable angina pectoris	21	IVW	1.000 (0.999–1.002)	0.62
			MR Egger	1.000 (0.999–1.002)	0.62
PD-1	Coronary atherosclerosis	21	IVW	0.999 (0.996–1.001)	0.32
			MR Egger	0.997 (0.993–1.002)	0.22
PD-L1	Coronary atherosclerosis	21	IVW	0.998 (0.994–1.001)	0.23
			MR Egger	0.998 (0.994–1.002)	0.44

IVW, inverse variance weighted; N SNP, number of genetic instruments; OR, odds ratio; CI, confidence interval.

*P < 0.05.

### 3.3 Bidirectional MR (effect of CHD on Pd-1/Pd-L1)

Chronic ischemic heart disease was shown by IVW to be significantly associated with PD-1 (beta, −3.1; 95%CI, −6.017 to −0.183, P, 0.037) and PD-L1(beta, −3.269; 95%CI, −6.197 to −0.341; P, 0.029) (Tables 4, 5). This result was consistent with other sensitivity analysis. The causal relationships between the other 4 CHDs and PD-1/PD-L1 were not statistically significant (Tables 4, 5). No outlier between CHD and PD-1/PD-L1 was identified by the MR-PRESSO test (Tables 4, 5). No directional pleiotropy was found in the MR-Egger regression (Tables 4, 5). No heterogeneity was found by Cochran's Q statistic (Tables 4, 5). The scatter plot and leave-one-out plot for the effects of 5 CHD on PD-1/PD-L1 are available in Figure 1, Supplementary Figures S3–S6, S9–S13. The selected SNPs were listed in Supplementary Table S17–S26.

**Table 4:** Single-Variable MR results of risk of chronic ischemic heart disease, acute myocardial infarction, angina pectoris, unstable angina pectoris and coronary atherosclerosis on PD-1.

Exposure	SNPS	Methods	Beta (95% CI)	*P*-value	Q-statistics	*P*h	*P*p
Chronic ischemic heart disease	50	IVW	−3.100 (−6.017 to −0.183)	0.04	46.150	0.59	
		MR Egger	−1.384 (−7.645 to 4.876)	0.67	45.782	0.56	0.55
		RAPS	−2.648 (−5.709 to 0.413)	0.09			
		Weighted median	−2.328 (−6.635 to 1.978)	0.29			
		MR Presso	NA	NA			
Acute myocardial infarction	21	IVW	−1.415 (−9.592 to 6.762)	0.73	22.354	0.32	
		MR Egger	−1.032 (−18.413 to 16.349)	0.91	22.352	0.27	0.96
		RAPS	−2.474 (−10.765 to 5.818)	0.56			
		Weighted median	1.706 (−9.363 to 12.775)	0.76			
		MR Presso	NA	NA			
Angina pectoris	23	IVW	3.203 (−3.823 to 10.23)	0.37	16.525	0.79	
		MR Egger	8.315 (−6.240 to 22.871)	0.28	15.907	0.77	0.44
		RAPS	3.197 (−4.267 to 10.661)	0.40			
		Weighted median	1.643 (−8.318 to 11.605)	0.75			
		MR Presso	NA	NA			
Unstable angina pectoris	20	IVW	4.999 (−4.446 to 14.445)	0.30	16.612	0.62	
		MR Egger	6.449 (−13.764 to 26.662)	0.54	16.587	0.55	0.88
		RAPS	5.710 (−5.177 to 16.597)	0.30			
		Weighted median	5.082 (−7.763 to 17.927)	0.44			
		MR Presso	NA	NA			
Coronary atherosclerosis	82	IVW	−0.912 (−2.905 to 1.081)	0.37	87.717	0.29	
		MR Egger	−3.169 (−7.594 to 1.256)	0.16	86.366	0.29	0.27
		RAPS	−0.676 (−2.692 to 1.340)	0.51			
		Weighted median	0.230 (−2.658 to 3.118)	0.88			
		MR Presso	NA	NA			

IVW, inverse variance weighted; RAPS, robust adjusted profile score; MR Presso, Mendelian randomization pleiotropy RESidual sum and outlier; N SNP, number of genetic instruments; OR, odds ratio; CI, confidence interval; *P*h, *P*-value for heterogeneity; *P*p, *P*-value for Pleiotropy; NA, not applicable.

**Table 5:** Single-Variable MR results of risk of chronic ischemic heart disease, acute myocardial infarction, angina pectoris, unstable angina pectoris and coronary atherosclerosis on PD-L1.

Exposure	SNPS	Methods	Beta (95% CI)	*P*-value	Q-statistics	*P*h	*P*p
Chronic ischemic heart disease	50	IVW	−3.269 (−6.197 to −0.341)	0.03	49.362	0.46	
		MR Egger	−4.008 (−10.352 to 2.337)	0.22	49.294	0.42	0.80
		RAPS	−3.563 (−6.685 to −0.441)	0.03			
		Weighted median	−3.615 (−8.136 to 0.907)	0.12			
		MR Presso	NA	NA			
Acute myocardial infarction	21	IVW	−1.775 (−9.509 to 5.959)	0.65	16.186	0.71	
		MR Egger	−11.635 (−27.658 to 4.389)	0.17	14.290	0.77	0.18
		RAPS	−0.771 (−8.951 to 7.409)	0.85			
		Weighted median	−1.314 (−11.928 to 9.300)	0.81			
		MR Presso	NA	NA			
Angina pectoris	23	IVW	−4.750 (−11.928 to 2.429)	0.19	22.951	0.40	
		MR Egger	−0.972 (−16.079 to 14.135)	0.90	22.614	0.36	0.58
		RAPS	−4.502 (−11.996 to 2.993)	0.24			
		Weighted median	−2.394 (−11.921 to 7.133)	0.62			
		MR Presso	NA	NA			
Unstable angina pectoris	20	IVW	0.356 (−10.754 to 11.467)	0.95	26.302	0.12	
		MR Egger	2.445 (−21.958 to 26.848)	0.85	26.249	0.09	0.85
		RAPS	−1.764 (−13.321 to 9.793)	0.76			
		Weighted median	−5.880 (−19.714 to 7.954)	0.40			
		MR Presso	NA	NA			
Coronary atherosclerosis	82	IVW	−0.694 (−2.703 to 1.315)	0.50	89.168	0.25	
		MR Egger	−3.354 (−7.802 to 1.095)	0.14	87.289	0.27	0.19
		RAPS	−1.084 (−3.146 to 0.979)	0.30			
		Weighted median	−2.607 (−5.756 to 0.542)	0.10			
		MR Presso	NA	NA			

IVW, inverse variance weighted; RAPS, robust adjusted profile score; MR Presso, Mendelian randomization pleiotropy RESidual sum and Outlier; N SNP, number of genetic instruments; OR, odds ratio; CI, confidence interval; *P*h, *P*-value for heterogeneity; *P*p, *P*-value for Pleiotropy; NA, not applicable.

### 3.4 GSEA analysis

**Figure 2:**
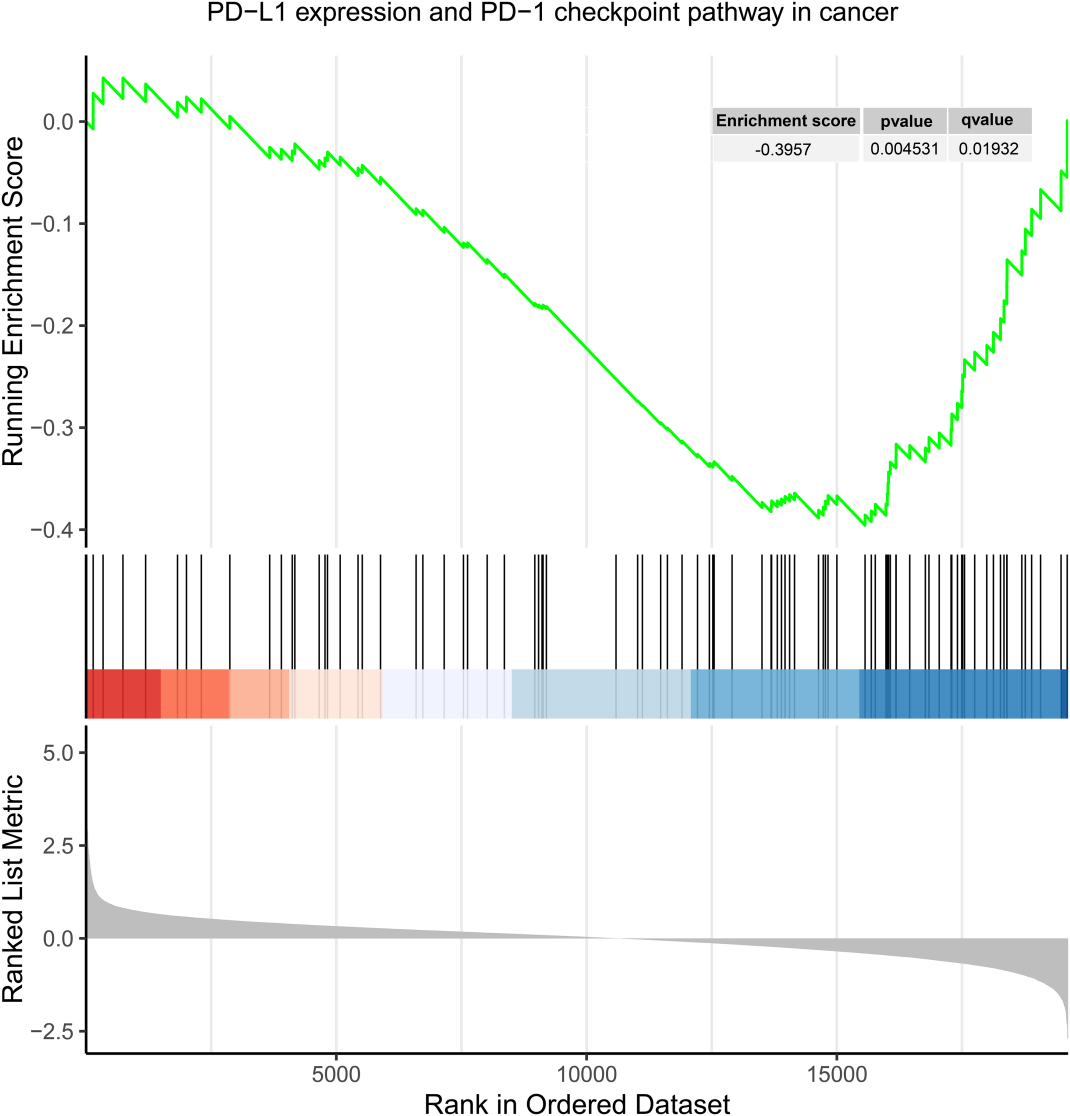
Enrichment plot for “PD-L1 expression and PD-1 checkpoint pathway in cancer” in GSE71226.

Gene sets (GSE71226) from both the control and CHD groups were screened under the conditions of an *q*-value < 0.25, and *P* < 0.05, resulting in 77 differentially expressed gene sets (Supplementary eTable S27). Among these 77 gene sets, the gene set of “PD-L1 expression and PD-1 checkpoint pathway in cancer” pathway was included and downregulated in CHD group compared to control group with an enrichment score of −0.396 (*P* = 0.005; *q* = 0.019) (Figure 2).

## 4 Discussion

This study revealed a protective causal relationship between PD-1 and chronic ischemic heart disease. In contrast, there was no evidence of a causal association between PD-1/PD-L1 and acute myocardial infarction, angina pectoris, unstable angina pectoris, or coronary atherosclerosis. After adjustment for PD-1 and PD-L1 through multivariable MR, the protective causal relationship between PD-1 and chronic ischemic heart disease remained. In reverse MR analysis, it was demonstrated that chronic ischemic heart disease was significantly associated with PD-1 and PD-L1 and no evidence was found in favor of association between other 4 CHD and PD-1 or PD-L1.

As well-characterized immune checkpoints, PD-1 and PD-L1 play a crucial role in the development of vascular inflammatory disorders by regulating T-cell activity (23, 24). Immune checkpoint inhibitors (ICIs) have become a well-established option for selected cancer therapies by conferring overall survival benefits to patients with advanced cancer, with an attendant focus on the cardiovascular adverse events that occur with ICI therapy. Since 2016, studies have reported cardiotoxicity due to ICI therapy (25, 26), PD-1/PD-L1 blockers provide a robust link to atherosclerosis, and lack of PD-1/PD-L1 exacerbates atherosclerotic patch formation by mechanisms that may be influenced through vascular inflammation, as well as T-cell activation and effector function (6, 11). PD-1 inhibitors can also induce cardiac injury via polarized Macrophages (27). This suggests a possible direct link between PD-1/PD-L1 itself for associated cardiovascular disease.

Previous studies demonstrated that patients with a history of myocardial infarction have significantly up-regulated PD-L1 expression (10). PD-L1 expression on peripheral blood T cells is significantly under-regulated in patients with coronary artery disease and might contribute to the pathogenesis and development of atherosclerosis (28). Fujisue et al. (29) proved that soluble PD-L1 (sPD-L1) levels were elevated in patients with coronary artery disease, and Miyazaki et al. (30) prospectively found elevated levels of sPD-L1 increased cardiovascular risk in these patients. Notably, a certain amount of circulating sPD-L1 acts to suppress the PD-1/PD-L1 pathway, thereby fostering chronic immune responses and inflammation, ultimately accelerating the pathological progression of CHD (31). The earlier studies, however, were mostly single-center and observed, failing to explain the causal relationship. Whereas to our knowledge there are no large randomized controlled trials assessing the causal link between PD-1/PD-L1 expression and CHD. Our study revealed a protective causal effect of PD-1 against chronic ischemic heart disease, a result that was held after multivariate analysis. Meanwhile, inverse MR also revealed that chronic ischemic heart disease was significantly associated with PD-1/PD-L1. The pathophysiological basis of chronic ischemic heart disease lies in coronary atherosclerosis, while the interaction of PD-1 and PD-L1 influences its onset and progression through various potential mechanisms. The PD-1/PD-L1 pathway restricts T cell activation, suppresses T cell responses, and promotes atherosclerosis. Simultaneously, PD-1 binding PD-L1 induces regulatory T cells, inhibiting the release of pro-inflammatory cytokines(e.g., IFN-*γ* and TNF-α), thereby aiding in reducing plaque volume. In addition, high PD-L1 expression diminishes T cell-mediated endothelial cytolytic damage, reducing vascular injury (11, 32, 33). Targeting the PD-1/PD-L1 pathway provides a novel perspective for prevention and treatment of chronic ischemic heart disease. Future research should delve into the intricate mechanisms of the PD-1/PD-L1 axis to optimize therapeutic or preventive strategies for chronic ischemic heart disease.

For other types of CHD, there is no clear causal relationship with PD-1/PD-L1, conceivably for the relevant genetic markers actually cause a broader susceptibility to coronary artery disease rather than being determinants, but the specific mechanisms need to be further explored. Only some subtypes of CHD are correlated with PD-1/PD-L1, possibly due to a combination of factors such as variations in the pathophysiological mechanisms of different disease subtypes and genetic influences. Firstly, each subtype of CHD exhibits distinct pathophysiological mechanisms (34, 35). Chronic ischemic heart disease may be more linked to chronic vascular inflammation, endothelial dysfunction, and atherosclerotic plaque formation, whereas acute myocardial infarction may be more associated with plaque rupture, thrombus formation, and acute coronary artery occlusion. The PD-1/PD-L1 signaling pathway plays a crucial role in immune regulation, potentially reducing inflammation by inhibiting T-cell activity (32). This effect might act as a protective mechanism in chronic ischemic heart disease, reducing ongoing vascular wall damage and plaque progression. However, in acute coronary events, this inhibitory effect may not be the primary pathological process, possibly overshadowed by more urgent physiological responses. Furthermore, the polymorphism of PD-1/PD-L1 genes may exert varying effects in different disease subtypes (36, 37). The interaction between genetic variations and environmental factors may also influence the role of PD-1/PD-L1 across different disease subtypes (38). Future research focusing on elucidating the specific mechanisms of the PD-1/PD-L1 signaling pathway in different CHD subtypes will contribute to a more accurate understanding of its causal relationships.

The current investigation offered several notable strengths. Initially, we delved into the reciprocal causal link between PD-1/PD-L1 and 5 types of CHD utilizing a bidirectional MR framework, thereby mitigating concerns regarding confounding factors, reverse causation, and exposure biases (39). Additionally, sensitivity assessments including MR-Egger, weighted median, and MR-PRESSO were conducted to bolster the consistency and resilience of our findings. Moreover, the integration of multivariable MR to adjust for confounding variables enhanced the reliability of inferring causal connections between PD-1/PD-L1 and CHD. Last, an additional validation of GSEA analysis was conducted to check whether CHD is associated with genes related to PD-1/PD-L1 pathway, increasing the rigorness of study.

Notwithstanding these strengths, it's essential to acknowledge certain limitations. Initially, a limited number of SNPs met the conventional bioinformatic threshold of *p* < 5 × 10^−8^. This scarcity of SNPs could pose challenges in matching instrumental variables (IVs) to the outcome, potentially weakening associations. Therefore, we opted for SNPs identified using a less stringent significance level of 5 × 10^−6^, a method recommended in prior research (40, 41). However, it's important to acknowledge that this approach may introduce a degree of weak instrumental variable bias. To assess this risk, we calculated F statistics, finding no substantial evidence supporting the presence of such bias (All IVs showed an *F* value greater than 10). Furthermore, our study focused on the European population, thus caution is warranted when generalizing our results to other demographic groups. Last, due to the aggregate nature of the GWAS data utilized in this study, we lacked access to data stratified by sex and age or individual-level data. Consequently, our investigation was unable to explore the potential causal associations between PD-1/PD-L1 and CHD across various age and sex subgroups.

## 5 Conclusion

This MR study supported a bidirectional causal relationship between PD-1 and chronic ischemic heart disease and protective association between chronic ischemic heart disease and PD-L1. Gene set related to PD-1/PD-L1 was revealed downregulated in CHD by GSEA, which consolidate the MR result. Though our results did not support a causal association between PD-1/PD-L1 and acute myocardial infarction, angina pectoris, coronary atherosclerosis, as well as unstable angina pectoris, further investigations are needed to clarify the mutual effect between PD-1/PD-L1 and CHD, as well as predictive models that detect intricate relationships and interactions among genetic variations, environmental influences, and the risk of CHD.

## Data Availability

The original contributions presented in the study are included in the article/Supplementary Material, further inquiries can be directed to the corresponding authors.
