# Supplementary material for: PD-1/PD-L1 and coronary heart disease: a mendelian randomization study
Source: Front Cardiovasc Med. 2024 Oct 18;11:1424770. doi: 10.3389/fcvm.2024.1424770 (PMC11527656; doi:10.3389/fcvm.2024.1424770)
Supplement: Supplementary file 2 [file Datasheet2.pdf]

Supplementary Material

1 Supplementary Figures and Tables

1.1 Supplementary Figures

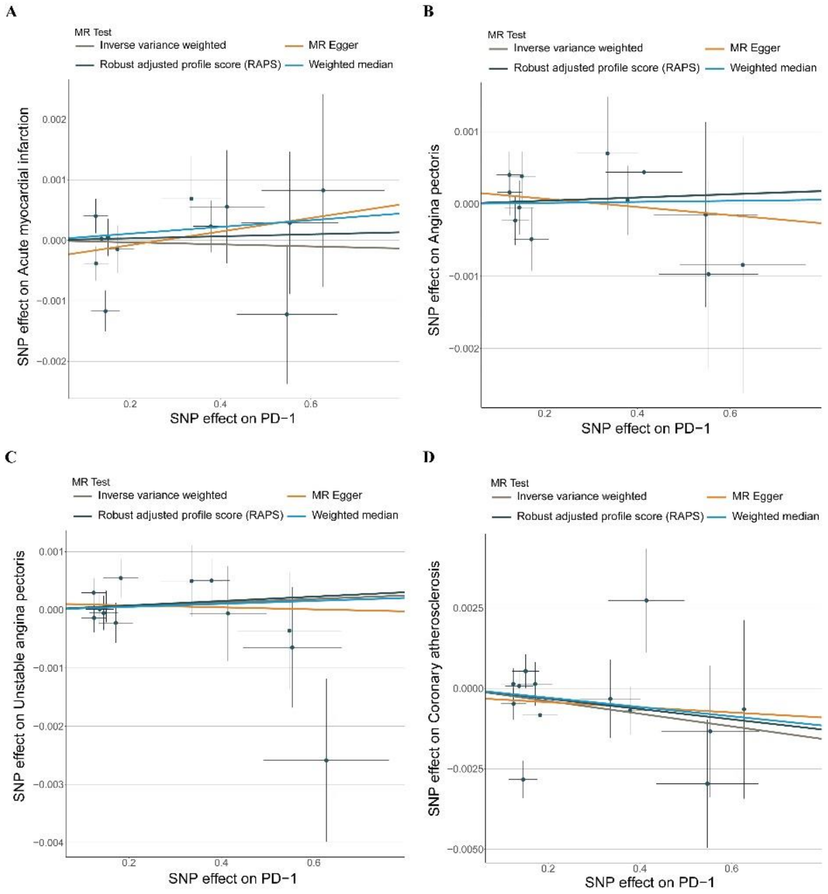

Figure S1: Scatter plot for PD-1 effects on acute myocardial infarction, angina pectoris, unstable angina pectoris, and coronary atherosclerosis;

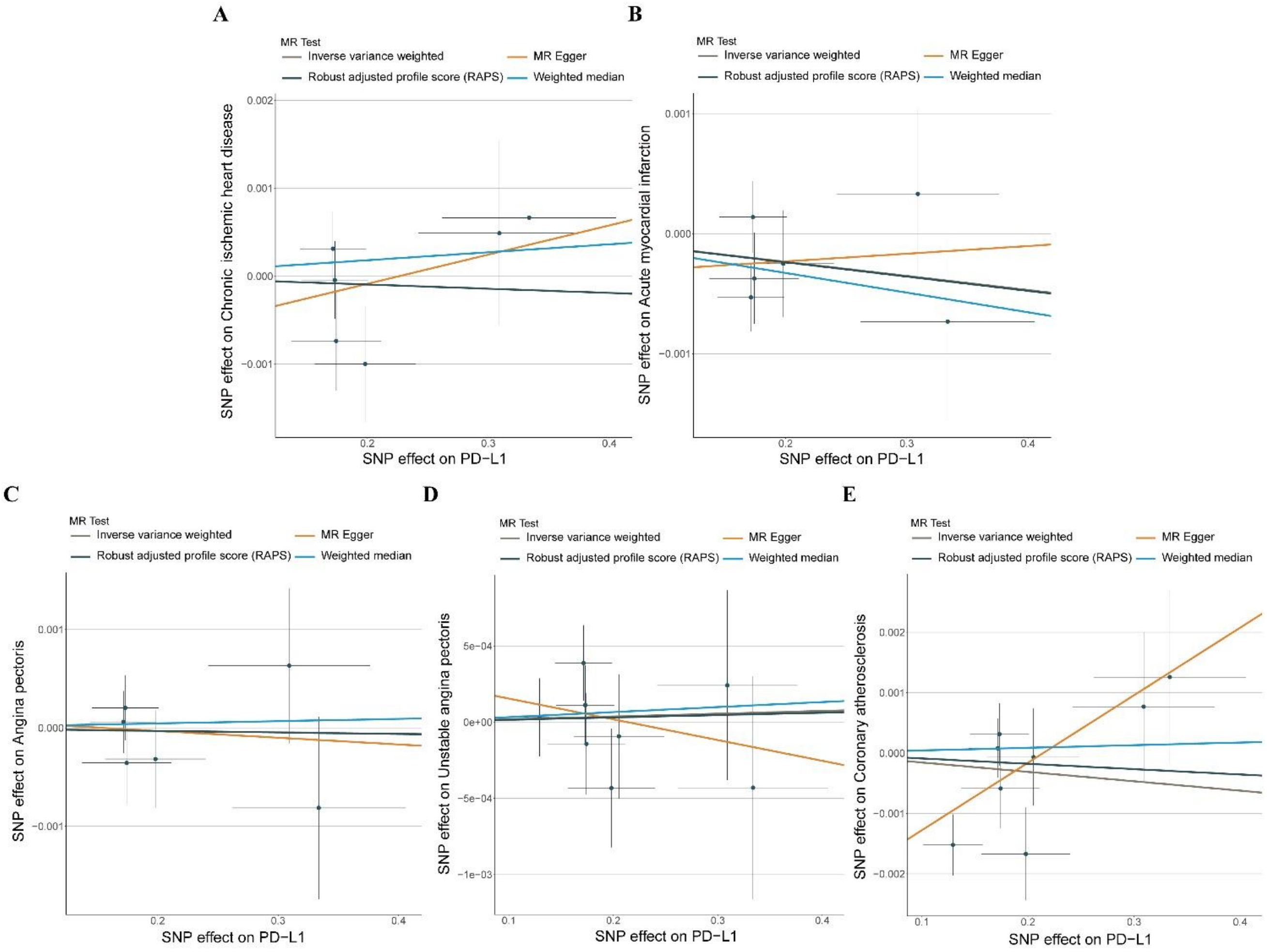

Figure S2: Scatter plot for PD-L1 effects on chronic ischemic heart disease, acute myocardial infarction, angina pectoris, unstable angina pectoris, and coronary atherosclerosis;

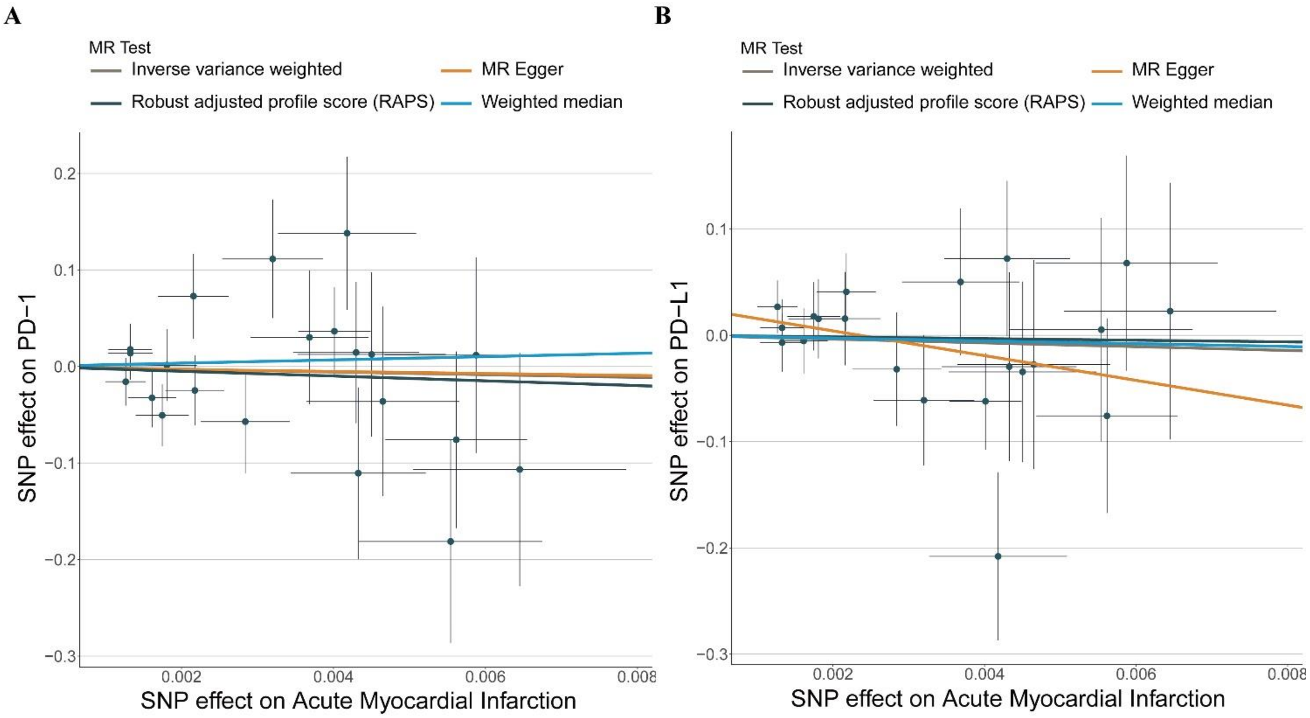

Figure S3: Scatter plot for the effects of acute myocardial infarction on PD-1/PD-L1;

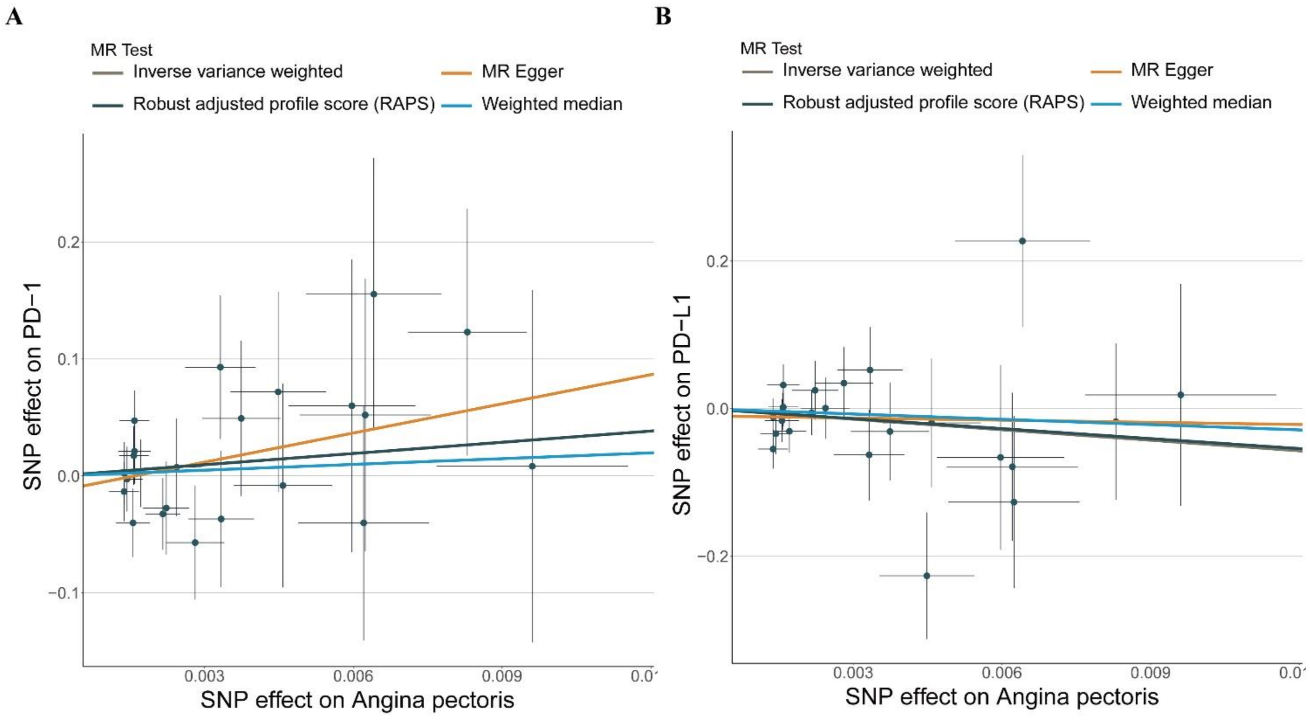

Figure S4: Scatter plot for the effects of angina pectoris on PD-1/PD-L1;

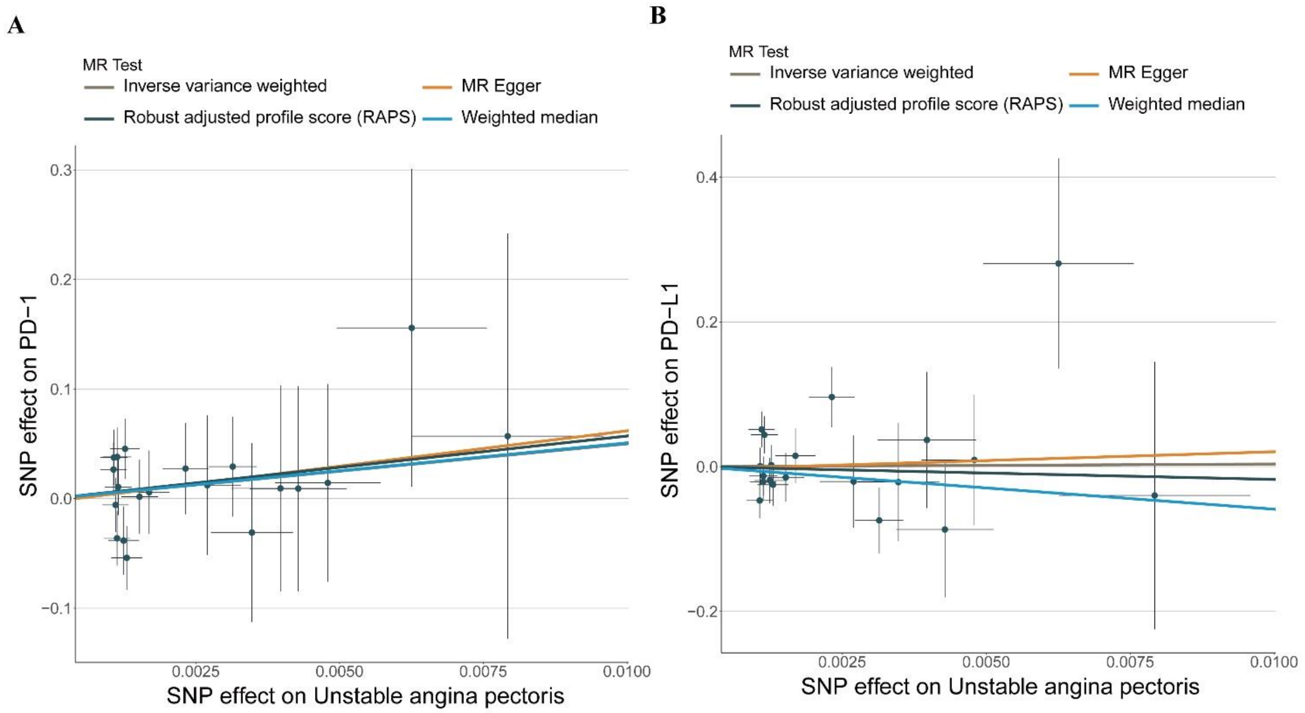

Figure S5: Scatter plot for the effects of unstable angina pectoris on PD-1/PD-L1;

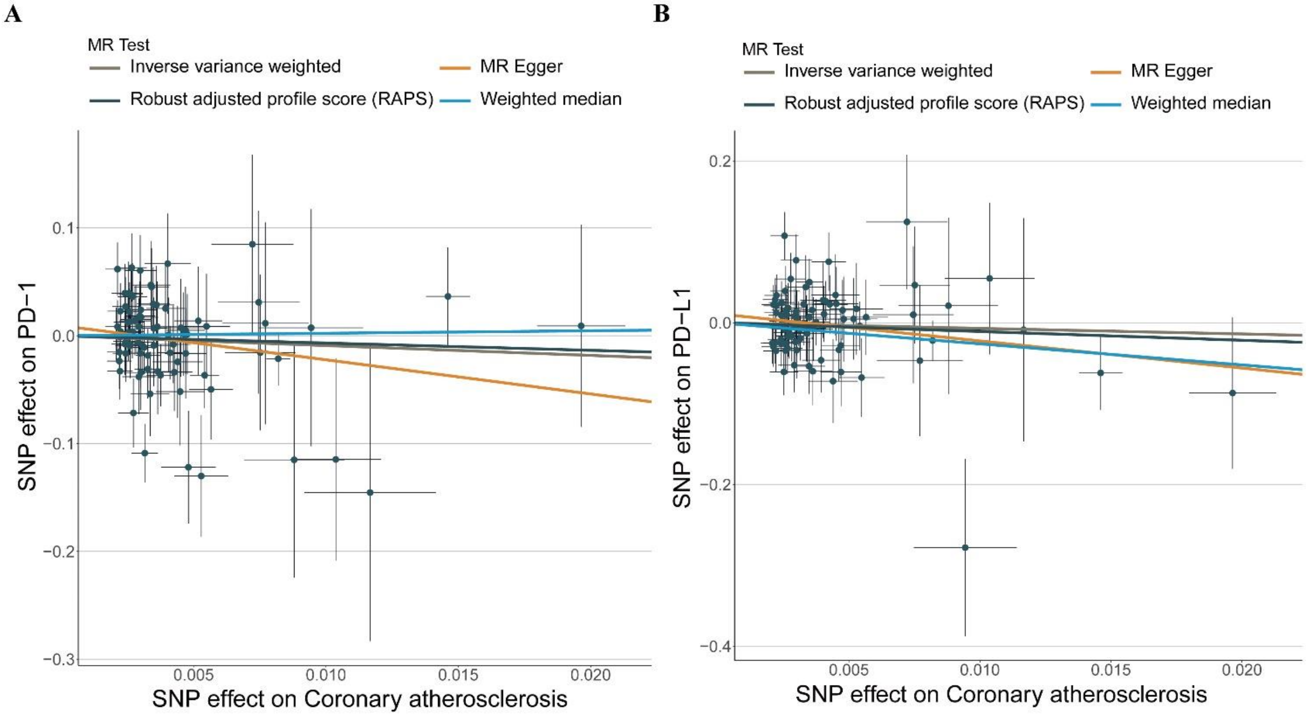

Figure S6: Scatter plot for the effects of coronary atherosclerosis on PD-1/PD-L1;

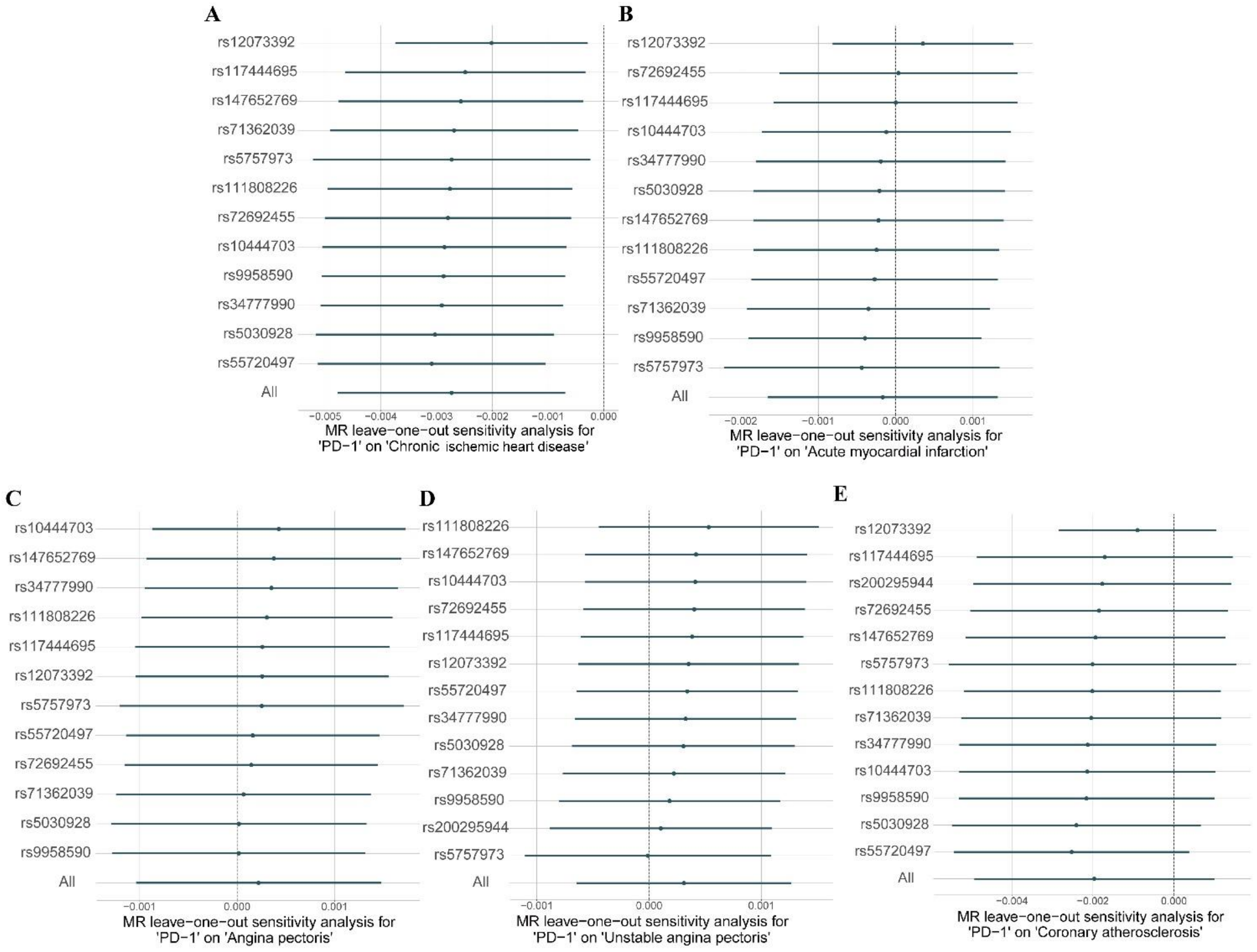

Figure S7: Leave-one-out plot for PD-1 effects on acute myocardial infarction, angina pectoris, unstable angina pectoris, and coronary atherosclerosis;

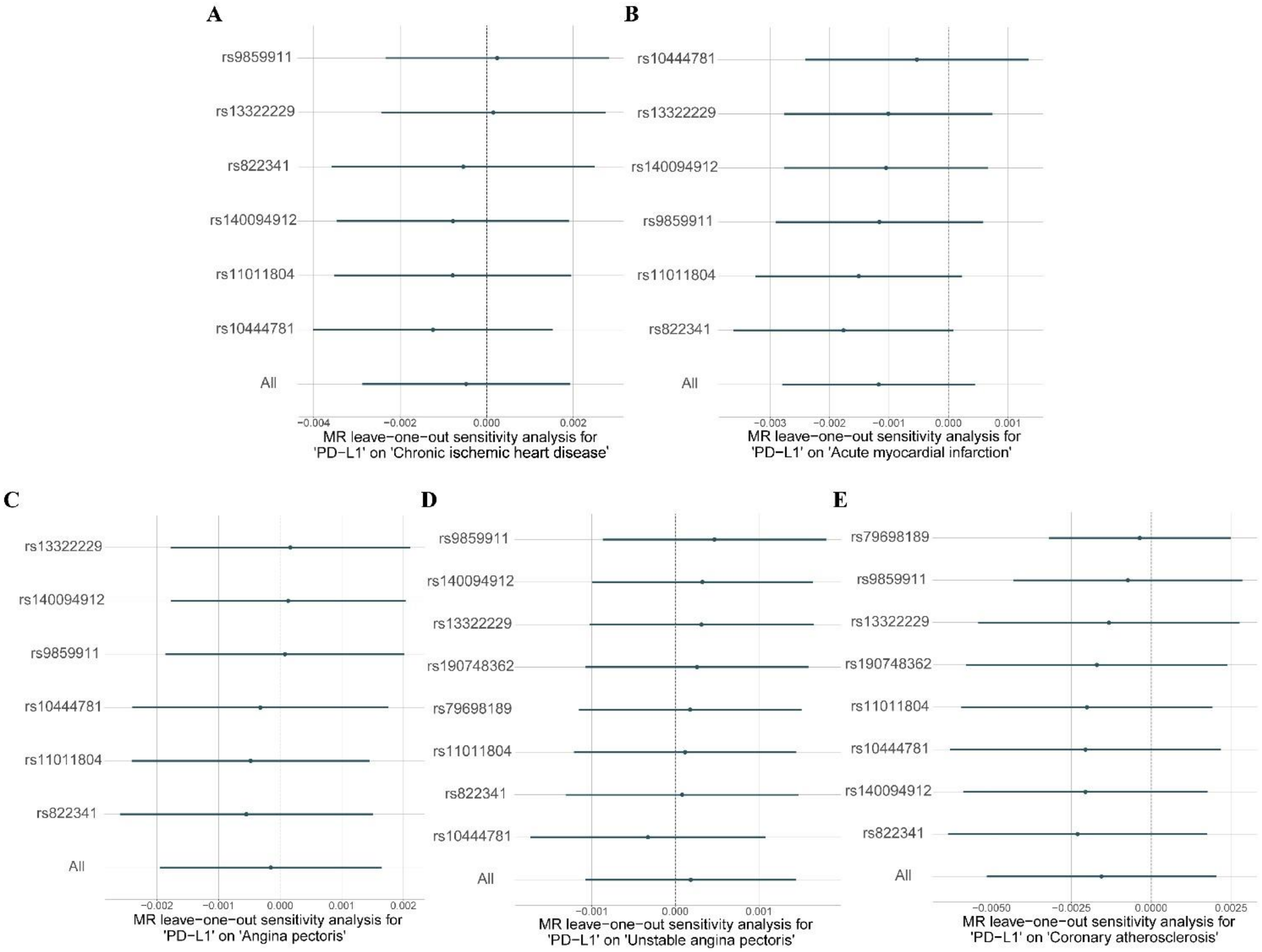

Figure S8: Leave-one-out plot for PD-1 effects on acute myocardial infarction, angina pectoris, unstable angina pectoris, and coronary atherosclerosis;

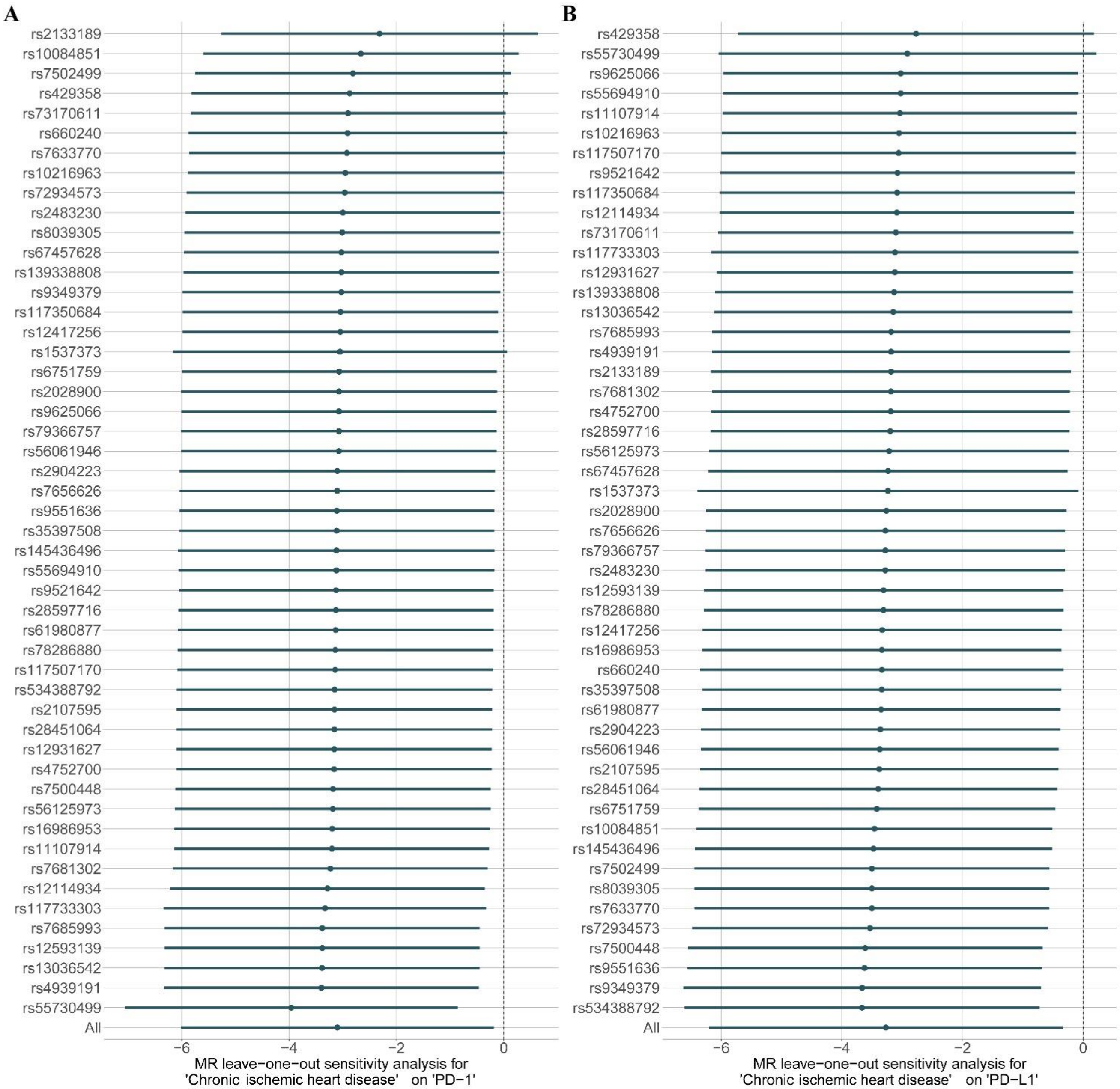

Figure S9: Leave-one-out plot for the effects of chronic ischemic heart disease on PD-1/PD-L1;

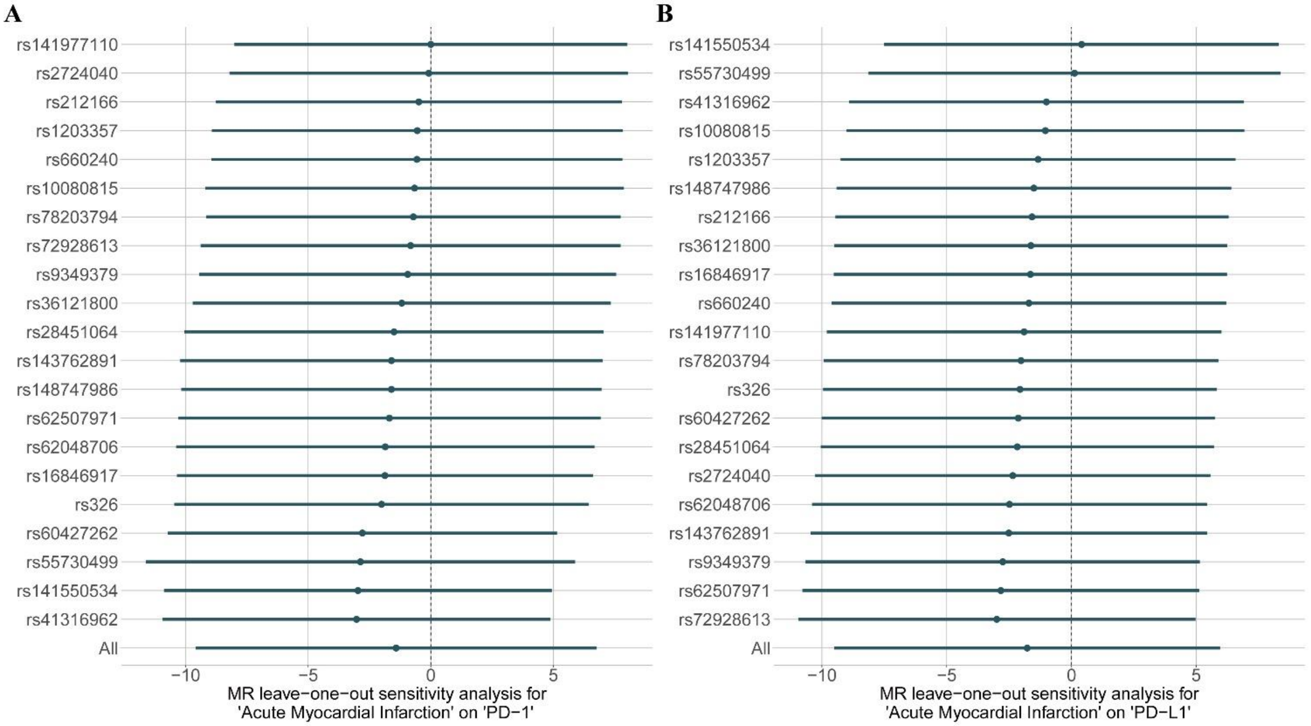

Figure S10: Leave-one-out plot for the effects of acute myocardial infarction on PD-1/PD-L1;

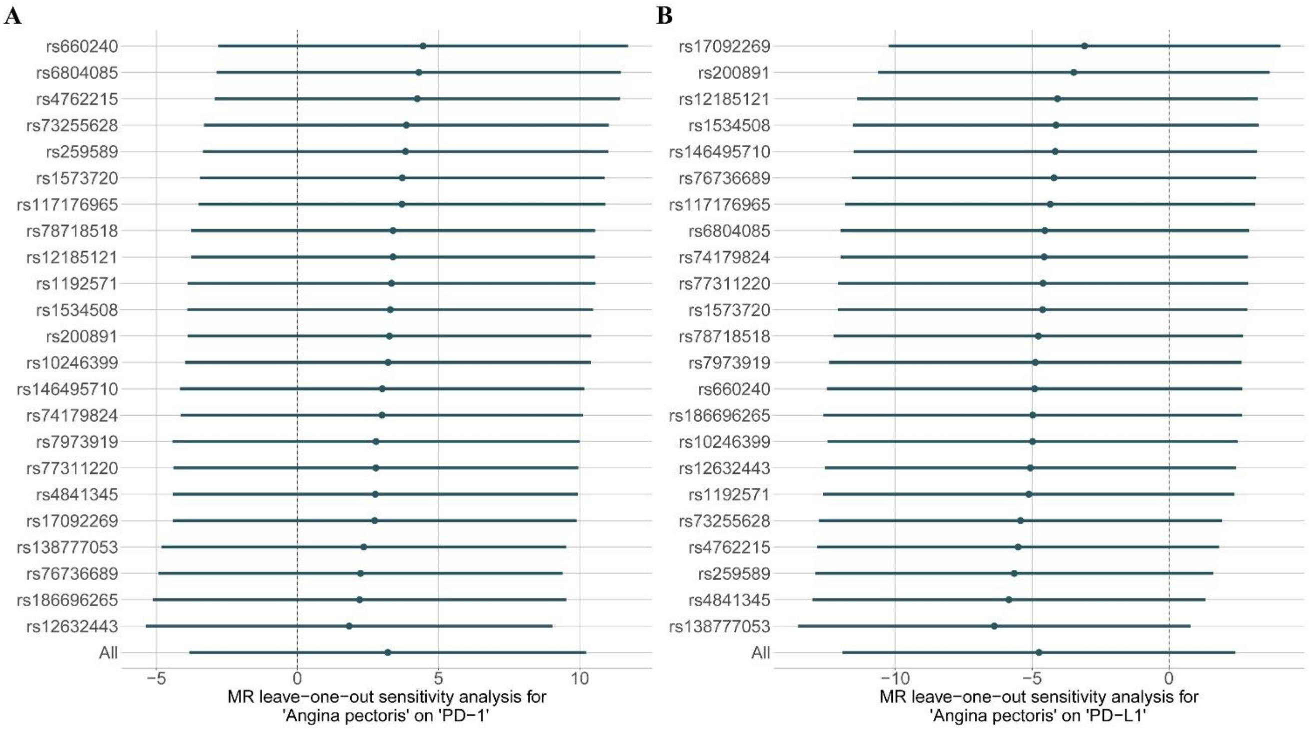

Figure S11: Leave-one-out plot for the effects of angina pectoris on PD-1/PD-L1;

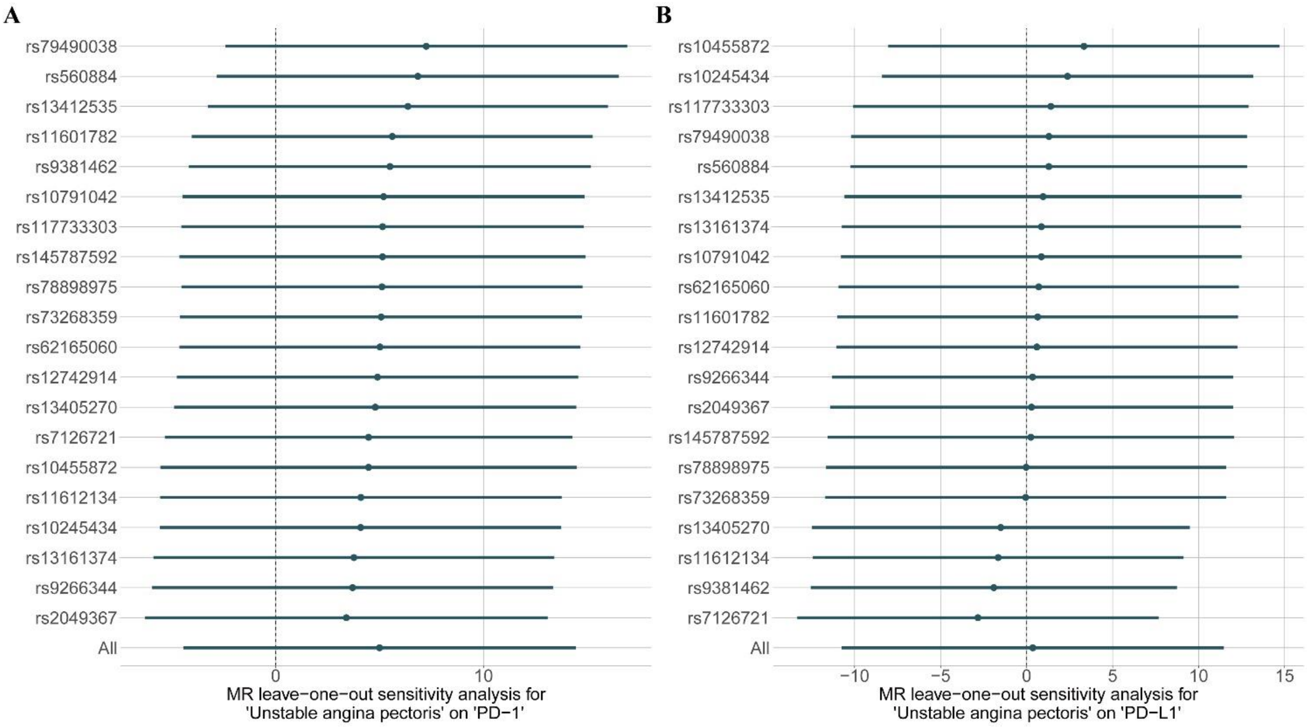

Figure S12: Leave-one-out plot for the effects of unstable angina pectoris on PD-1/PD-L1;

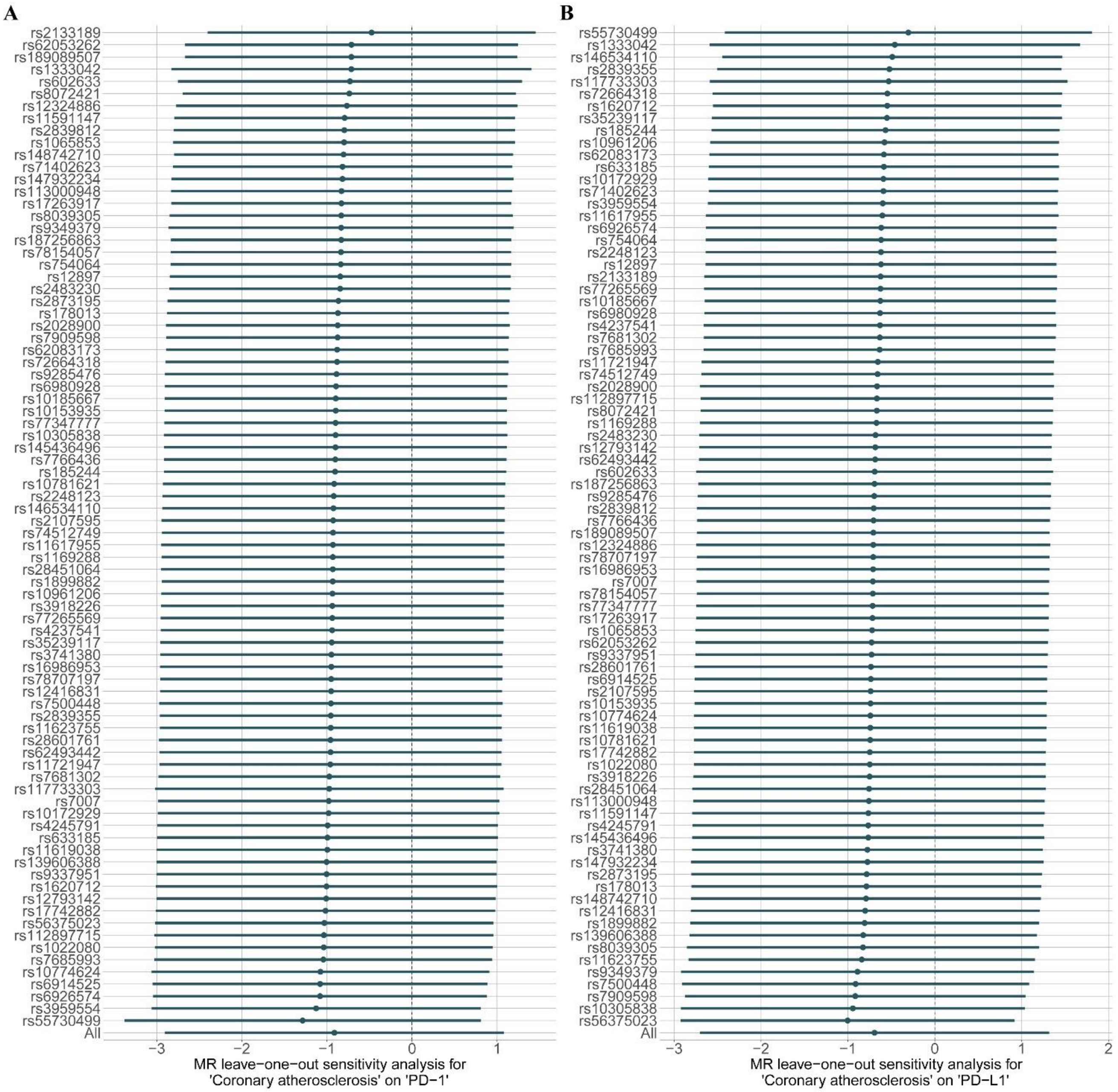

Figure S13: Leave-one-out plot for the effects of coronary atherosclerosis on PD-1/PD-L1.

1.2 Supplementary Tables

Table S1: PD-1, PD-L1， Chronic ischemic heart disease, Acute myocardial infarction, Angina pectoris Use Phenotypes, Coronary atherosclerosis and Unstable angina pectoris: Source and Description (see separated Excel table);

Table S2: Independent Instruments for PD-1 Use in Chronic ischemic heart disease: Harmonized Data (see separated Excel table);

Table S3: Independent Instruments for PD-1 Use in Acute myocardial infarction: Harmonized Data (see separated Excel table);

Table S4: Independent Instruments for PD-1 Use in Angina pectoris: Harmonized Data (see separated Excel table);

Table S5: Independent Instruments for PD-1 Use in Unstable angina pectoris: Harmonized Data (see separated Excel table);

Table S6: Independent Instruments for PD-1 Use in Coronary atherosclerosis: Harmonized Data (see separated Excel table);

Table S7: Independent Instruments for PD-L1 Use in Chronic ischemic heart disease: Harmonized Data (see separated Excel table);

Table S8: Independent Instruments for PD-L1 Use in Acute myocardial infarction: Harmonized Data (see separated Excel table);

Table S9: Independent Instruments for PD-L1 Use in Angina pectoris: Harmonized Data (see separated Excel table);

Table S10: Independent Instruments for PD-L1 Use in Unstable angina pectoris: Harmonized Data (see separated Excel table);

Table S11: Independent Instruments for PD-L1 Use in Coronary atherosclerosis: Harmonized Data (see separated Excel table);

Table S12: Multivariable Instruments for Exposures PD-1 and PD-L1 on Chronic ischemic heart disease: Harmonized Data (see separated Excel table);

Table S13: Multivariable Instruments for Exposures PD-1 and PD-L1 on Acute myocardial infarction: Harmonized Data (see separated Excel table);

Table S14: Multivariable Instruments for Exposures PD-1 and PD-L1 on Angina pectoris: Harmonized Data (see separated Excel table);

Table S15: Multivariable Instruments for Exposures PD-1 and PD-L1 on Unstable angina pectoris: Harmonized Data (see separated Excel table);

Table S16: Multivariable Instruments for Exposures PD-1 and PD-L1 on Coronary atherosclerosis: Harmonized Data (see separated Excel table);

Table S17: Independent Instruments for Chronic ischemic heart disease Use in PD-1: Harmonized Data (see separated Excel table);

Table S18: Independent Instruments for Acute myocardial infarction Use in PD-1: Harmonized Data (see separated Excel table);

Table S19: Independent Instruments for Angina pectoris Use in PD-1: Harmonized Data (see separated Excel table);

Table S20: Independent Instruments for Unstable angina pectoris Use in PD-1: Harmonized Data (see separated Excel table);

Table S21: Independent Instruments for Coronary atherosclerosis Use in PD-1: Harmonized Data (see separated Excel table);

Table S22: Independent Instruments for Chronic ischemic heart disease Use in PD-L1: Harmonized Data (see separated Excel table);

Table S23: Independent Instruments for Acute myocardial infarction Use in PD-L1: Harmonized Data (see separated Excel table);

Table S24: Independent Instruments for Angina pectoris Use in PD-L1: Harmonized Data (see separated Excel table);

Table S25: Independent Instruments for Unstable angina pectoris Use in PD-L1: Harmonized Data; Table S26: Independent Instruments for Coronary atherosclerosis Use in PD-1: Harmonized Data (see separated Excel table).
